# Hydrogen attenuates postoperative pain through Trx1/ASK1/MMP9 signaling pathway

**DOI:** 10.1186/s12974-022-02670-0

**Published:** 2023-02-03

**Authors:** Juan Li, Shirong Ruan, Jinhui Jia, Qian Li, Rumeng Jia, Li Wan, Xing Yang, Peng Teng, Qilin Peng, Ya-dan Shi, Pan Yu, Yinbing Pan, Man-lin Duan, Wen-Tao Liu, Li Zhang, Liang Hu

**Affiliations:** 1 Department of Anesthesiology, BenQ Medical Center, The Affiliated BenQ Hospital of Nanjing Medical University, Nanjing, 210019 Jiangsu China; 2 Department of Pharmacology, School of Basic Medical Sciences, Nanjing Medical University, Nanjing, 210029 Jiangsu China; 3 Department of Orthopedics, Jiangsu Province Hospital of Integration of Chinese and Western Medicine, Nanjing, 210029 Jiangsu China; 4 Department of Anesthesiology, The Affiliated Jiangning Hospital of Nanjing Medical University, Nanjing, 211100 Jiangsu China; 5 Department of Pathology, The Affiliated Jiangning Hospital of Nanjing Medical University, Nanjing, 211100 Jiangsu China; 6 Department of Burn and Plastic, Jingling Hospital, School of Medicine, Nanjing University, Nanjing, 210002 Jiangsu China; 7 Department of Anesthesiology, Children’s Hospital of Nanjing Medical University, 72 Guangzhou Road, Nanjing, 210008 Jiangsu China; 8 Department of Anesthesiology, The First Affiliated Hospital of Nanjing Medical University, Nanjing, 210029 China; 9 Department of Anesthesiology, Affiliated Jinling Hospital, Medical School of Nanjing University, Nanjing, 210002 Jiangsu China; 10 State Key Laboratory of Pharmaceutical Biotechnology, Nanjing University, Nanjing, 210002 Jiangsu China

**Keywords:** Postoperative pain, H_2_, MMP-9, ASK1

## Abstract

**Background:**

Postoperative pain is a serious clinical problem with a poorly understood mechanism, and lacks effective treatment. Hydrogen (H_2_) can reduce neuroinflammation; therefore, we hypothesize that H_2_ may alleviate postoperative pain, and aimed to investigate the underlying mechanism.

**Methods:**

Mice were used to establish a postoperative pain model using plantar incision surgery. Mechanical allodynia was measured using the von Frey test. Cell signaling was assayed using gelatin zymography, western blotting, immunohistochemistry, and immunofluorescence staining. Animals or BV-2 cells were received with/without ASK1 and Trx1 inhibitors to investigate the effects of H_2_ on microglia.

**Results:**

Plantar incision surgery increased MMP-9 activity and ASK1 phosphorylation in the spinal cord of mice. MMP-9 knockout and the ASK1 inhibitor, NQDI-1, attenuated postoperative pain. H_2_ increased the expression of Trx1 in the spinal cord and in BV-2 cells. H_2_ treatment mimicked NQDI1 in decreasing the phosphorylation of ASK1, p38 and JNK. It also reduced MMP-9 activity, downregulated pro-IL-1β maturation and IBA-1 expression in the spinal cord of mice, and ameliorated postoperative pain. The protective effects of H_2_ were abolished by the Trx1 inhibitor, PX12. In vitro, in BV-2 cells, H_2_ also mimicked NQDI1 in inhibiting the phosphorylation of ASK1, p38, and JNK, and also reduced MMP-9 activity and decreased IBA-1 expression induced by LPS. The Trx1 inhibitor, PX12, abolished the protective effects of H_2_ in BV-2 cells.

**Conclusions:**

For the first time, the results of our study confirm that H_2_ can be used as a therapeutic agent to alleviate postoperative pain through the Trx1/ASK1/MMP9 signaling pathway. MMP-9 and ASK1 may be the target molecules for relieving postoperative pain.

## Background

Postoperative pain is the most common health problem caused by tissue and nerve lesions after surgical injury. Approximately 80% of patients who experience surgical injury have acute pain postoperatively, and this progresses to severe chronic pain in over 20% of patients [1, 2]. However, effective treatments for postoperative pain are still scarce [3–5].

Neuroinflammation mediated by glia, especially microglia, induces central sensitization and plays a crucial role in the pathogenesis of pain [4]. Studies have shown that matrix metalloproteinases (MMPs) represent a novel mechanism and potential therapeutic target for neuroinflammation and pain [6–8]. The main MMPs involved in the generation and maintenance of pain include MMP-2 and MMP-9. After nerve injury, a rapid and transient increase in MMP-9 in early activated microglia by the cleavage of IL-1β and the activation of p38 induces neuropathic pain, while MMP-2 produces a delayed increase in maintaining neuropathic pain [8, 9]. Further studies have shown that MMP-9 inhibition suppresses astrocyte and microglia activation, and thus alleviates pain, while MMP-9 knockout significantly reduces neuralgia and tolerance to morphine [8–10]. Our previous study showed that paeoniflorin inhibits MMP9/2 and suppresses postoperative pain induced by plantar incisions [11]. Therefore, inhibition of MMP9-induced neuroinflammation may be a novel approach for the treatment of postoperative pain.

We explored signaling molecules upstream of MMP-9 to identify key targets for the inhibition of neuroinflammation. Apoptosis signal-regulating kinase 1 (ASK1), a member of the mitogen-activated protein kinase (MAP3K) family, activates JNK, MAPK, and p38 MAPK to induce the expression of MMP9 and amplify the inflammatory cascade; hence, it participates in the induction and maintenance of neuroinflammation [12, 13]. Meanwhile, there is a negative regulator, thioredoxin 1 (Trx1), in cells that inhibits ASK1 activity and maintains intracellular homeostasis. Trx1 interacts with the ASK1 region located between residues 46 and 277, and this interaction is believed to inhibit the activation of ASK1 by blocking its homo-oligomerization [14, 15]. However, whether the regulation of Trx1 on ASK1 can relieve postoperative pain has not been reported.

Our previous studies have shown that hydrogen (H_2_) alleviates lung injury during sepsis by upregulating Trx1 [16]. H_2_ has also been reported to inhibit MMP-9 activity in order to reduce brain infarction and improve neurological function [17]. H_2_ is a safe molecule with few adverse effects, and could pass through the blood–brain barrier and have protective roles in various pathological conditions, especially in cases of brain ischemia, reperfusion injury [18, 19], and inflammation [20, 21]. Therefore, we speculated that H_2_ may reduce neuroinflammation and alleviate postoperative pain, and examine whether the Trx1/ASK1 signaling pathway may be involved.

## Materials and methods

### Animals and ethics statement

The animal experiments were reviewed and approved by the animal care and use committee, and husbandry was strictly performed in line with the recommendations of the Guide for the Care and Use of Laboratory Animals (Ministry of Science and Technology of China, 2006) and the Nanjing Medical University Animal Care and Use Committee (NMJU-ACUC). During the experiments, pain and use of animals were minimized. Wild-type (WT, ICR/JCL, or C57BL/6) male mice (6–8 weeks) and MMP-9 knockout (C57BL/6) male mice (6–8 weeks) were approved by the Nanjing Medical University Animal Center. All animals were reared under pathogen-free conditions with controlled temperature (22 ± 2 °C) and a standardized light/dark cycle. They were given access to food and water ad libitum. All animals were allowed to acclimate to these conditions for approximately 1 week before being included in the experiments.

### Surgery

Plantar surgery was performed according to a previous study [22]. All surgeries were performed under anesthesia induced by isoflurane (2% oxygen gas, 300 mL/min). The plantar aspect of the left hind paw was sterilized with a 10% povidone-iodine solution before and after surgery, and placed through a hole in a sterile drape. A 0.8-cm longitudinal and 2-mm plutonic incision extending from the proximal edge of the heel to the toes was made through the skin and fascia of the plantar aspect of the foot. Then the skin was elevated and longitudinally incised through leaving the origins and insertions of the muscle intact. The skin was then apposed using 5-0 nylon sutures. The incision was checked daily and any subject whose plantar incision had signs of wound infection or dehiscence was excluded from the study.

### Drugs and reagents

NQDI-1 was purchased from Selleck Chemical Inc. (Houston, TX) (dissolved in 0.1% dimethyl sulfoxide [DMSO]). PX12 was purchased from MedChem Express (USA) (dissolved in DMSO). Antibodies for thioredoxin (#2429S), p-p38 (Thr180/Tyr182) (#9215S), p-JNK (Thr183/Tyr185) (#9255S), and p-ERK1/2 (Thr202/Tyr204) (#4377S) were purchased from Cell Signaling Technology (Beverly, MA). Antibodies for p-ASK1 (Thr845) (sc-109911) and β-actin (sc-4778) were purchased from Santa Cruz Biotechnology (Santa Cruz, CA, USA). Antibodies for IBA-1 (ab178847) and MMP-9 (ab58803) were purchased from Abcam (Cambridge, MA). The antibody for IL-1β was purchased from R&D systems (USA). Elisa kit (IL-1β, IL-6, TNF-α) were purchased from MULTI SCIENCES. Lipopolysaccharide (LPS) and DMSO were purchased from Sigma (St. Louis, MO, USA). All other chemicals were purchased from Sigma Chemical Co. (St. Louis, MO).

The procedures of saturated hydrogen-rich saline (HRS) were prepared as previously described [23]. They were prepared using H_2_ dissolved in normal saline for 12 h under high pressure (0.4 Mpa) and stored at 4 °C in an aluminum bag under normal pressure. To ensure that the hydrogen concentration was greater than 0.8 ppm, HRS was freshly prepared each time, and assessed using gas chromatography ENH-1000 (Trustiex Co, Japan) and Methylene Blue (Seo, Med Gas Res, Japan).

### Grouping and treatment

Mice were randomly assigned to the following groups: sham, plantar incision (PI), PI + HRS (5 mL/kg, i.p.); HRS treatment alone, PI + NDQI-1 (5 μg/10 μl, i.t.); and NDQI-1 treatment alone groups, PI + HRS + PX12 (12 μg/10 μl, i.t.). In the HRS treatment groups, the mice received injections twice daily, and in the NDQI-1 treatment groups, the mice received injections once daily. HRS or NDQI-1 were injected 6 h before plantar incision surgery. PX12 was injected 0.5 h before HRS administration. Each group consisted of 12 mice. The experimental groups and the timing of analysis are presented in Additional file 1: Table S1. BV-2 cells were kept in humidified 5% CO_2_ at 37 °C in Dulbecco’s modified Eagle’s medium supplemented with 10% FBS, penicillin (100 U/ml), and streptomycin (100 U/ml). A total of 10^5^ cells were seeded in 6-well plates overnight and divided into the following groups (*n* = 4/group): sham; LPS (1 μg/ml); LPS + H_2_ (5% CO_2_, 20% O_2_, 60% H_2_, PH-1-A, China); H_2_ alone; LPS + NQDI1 (10 μM); and LPS + H_2_ + PX12 (8 μM) groups. NQDI1 was added after LPS stimulation. PX12 was added 0.5 h before H_2_ treatment.

### Behavioral analysis

Before baseline testing, all animals were placed in the testing environment for at least 2 days for acclimatization. The first researcher was responsible for numbering the animal groups and drugs, as well as for data analysis. The second researcher administered the drugs to the groups according to the assigned numbers, while the third researcher performed the behavioral tests. The staff involved were blinded. Mechanical sensitivity was detected using von Frey hairs (Woodland Hills, Los Angeles, CA, USA). The animals were placed in boxes with an elevated metal mesh floor for habituation 30 min before testing. A series of von Frey hairs (the pressure intensity of mice between 0.16 g and 0.008 g) with logarithmically incrementing stiffness were used to perpendicularly stimulate the plantar surface of each hind paw. Each mouse was tested three times and the average of the threshold was measured.

Thermal hyperalgesia was detected using an analgesia meter (UGO Basile, Gemonio, Varese, Italy). Briefly, each mouse was placed on a 55 °C hot-plate apparatus. The heat source was focused on a portion of the hind paw, which was flush against the glass, and a radiant thermal stimulus was delivered to that site. The stimulus was then shut off when the hind paw moved (or after 20 s to prevent tissue damage). The intensity of the heat stimulus was kept constant throughout all the experiments. The elicited paw movement occurred at a latency of between 9 and 14 s in control animals. Thermal stimuli were delivered three times to each hind paw at 5- to 6-min intervals. Behavioral tests were performed blindly.

### Gelatin zymography

The lumbar spines (L4–L5) of mice were collected and analyzed at 24 h or 5 days after surgery. Mice were anesthetized, and spinal cord segments were rapidly dissected and homogenized in 1% Nonidet P-40 lysis buffer. The solubilized proteins were then resolved on gels (8% polyacrylamide gel containing 0.1% gelatin). After electrophoresis, each gel was incubated with 50 mL of developing buffer for 48 h (37.5 °C) in a shaking bath. Finally, the gels were stained with Coomassie Brilliant Blue (1%, with 10% acetic acid and 10% isopropyl alcohol, diluted with double-distilled H_2_O).

### Western blot analysis

Lumbar spine samples (L4–L5) were collected and analyzed at 24 h or 5 days after surgery. Cell samples were collected and analyzed 24 h after H_2_ treatment. Protein concentrations were determined by the BCA protein assay (Thermo Fisher Scientific, Waltham, MA, USA), and equal amounts of protein per lane were separated using 8–15% sodium dodecyl sulfate–polyacrylamide gel, and transferred to polyvinylidene fluoride membranes (Millipore Corp., Bedford, MA, USA). After being blocked with 5% bovine serum albumin and 5% skim milk for 2 h at room temperature, the membranes were incubated overnight at 4 °C with primary antibodies and then incubated with horseradish peroxidase (HRP)-conjugated secondary antibodies for 2 h at room temperature. The primary antibodies used included thioredoxin (1:500), p-p38 (1:1000), p-JNK (1:1000), p-ERK1/2 (1:1000), p-ASK1(1:1000), IL-1b (1:500), and IBA-1 (1:1000). For loading control, the blots were probed with an antibody for β-actin (1:5000). The filters were then developed by enhanced chemiluminescence reagents (PerkinElmer, Waltham, MA, USA) with secondary antibodies, Sigma (St. Louis, MO, USA). Finally, data were acquired using the Molecular Imager (Gel DocTM XR, 170–8170) and analyzed using the associated software Quantity One 4.6.5 (Bio-Rad Laboratories, Hercules, CA).

### Immunofluorescence staining

The lumbar spines (L4–L5) of the mice were collected and analyzed 5 days postoperatively. After deep anesthesia, the animal was perfused transcardially with normal saline followed by 4% paraformaldehyde. The lumbar segment L4 and/or L5 were dissected and postfixed in the same fixative. The embedded blocks were sectioned to a thickness of 20 μm. The sections of each group (six animals in each group) were then incubated with rabbit antibody for IBA-1 (1:100, #ab178846, abcam, Cambridge, MA, USA), mouse antibody for MMP-9 (1:100, #10375-2-AP, Proteintech, Rosemont, IL USA), secondary antibody (1:300, Alexa Fluor 488 AffiniPure Donkey Anti-Rabbit/Mouse IgG, #711–545-152/#715–545-150, Jackson ImmunoResearch Laboratories, USA) at room temperature. After being washed three times with PBS, all slides were processed blindly and then studied under a confocal microscope (Olympus FV1000 confocal system, Olympus, Japan) to observe morphological details after staining. Images were randomly coded and fluorescence intensities were analyzed using Image Pro Plus 6.0 software (Media Cybernetics Inc., Rockville, MD, USA). The average green and red fluorescence intensity of each pixel was normalized to the background intensity in the same image.

### Immunohistochemistry staining

The lumbar spines (L4–L5) of the mice were collected and analyzed at 24 h or 5 days after surgery. The samples were sectioned to 5 μm thickness, then incubated with the first antibodies for thioredoxin (1:100), mouse antibodies MMP-9 (1:100) and rabbit antibodies for IBA-1(1:300) in 10% donkey serum and 0.3% Triton-X100. After quenching endogenous peroxidase activity, the slides were washed in PBS and incubated with HRP conjugated secondary antibody for 2 h. Diaminobenzidine was used as a chromogen and counterstaining was performed with hematoxylin. Two independent pathologists evaluated all immunohistochemistry staining sections. The score for each slide was measured as the cross-product of the value of immunostaining intensity and the value of the proportion of positive-staining cells. The intensity of immunostaining was divided into four grades: 0, negative; 1, weak; 2, medium; 3, strong. The proportion of positive-staining cells was also divided into four grades: 1, 0 − 25%; 2, 26 − 50%; 3, 51 − 75%; and 4, > 75%. The score was calculated using the following formula: total score = intensity score × proportion score.

### Statistical analysis

GraphPad Prism 6 software (GraphPad Software, San Diego, CA, USA) was used to conduct all statistical analysis. Continuous variables were presented as means ± SEM. Normally distributed alteration of protein detected expression and changes in behavioral responses were tested with Student’s *t* tests and one-way ANOVA. The differences in latency over time among groups were tested with two-way ANOVA. Bonferroni post hoc comparisons was performed between multiple groups. A *criterion* value of *P* < 0.05 was considered statistically significant.

## Results

### MMP-9 participates in the development of plantar incision-induced postoperative pain

**Fig. 1:**
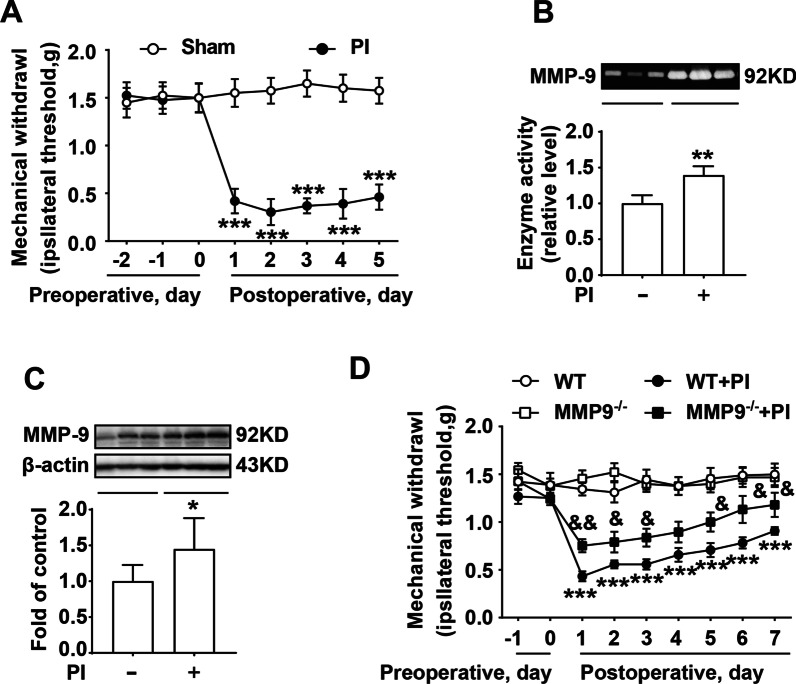
MMP-9 plays a crucial role in the development of plantar incision-induced postoperative pain. **A** Mechanical threshold of mice that received plantar incision surgery (*n* = 12). **B** MMP-9 activity was measured using zymography after surgery in the spinal cord of mice (*n* = 4). **C** MMP-9 expression in the spinal cord of mice was measured using western blot after surgery (*n* = 4). Lumbar spines (L4–L5) were collected and analyzed 5 days after surgery. **D** Mechanical threshold of wild-type or MMP-9 KO mice that underwent plantar incision surgery (*n* = 6). PI = plantar incision. Significant difference is revealed following two-way ANOVA (A and D) or unpaired Student’s t-test (**B**, **C**) (*P < 0.05, **P < 0.01, ***P < 0.001 vs. sham; ^&^*P* < 0.05, ^&&^*P* < 0.01 vs. PI group; Bonferroni post hoc tests)

To explore the role of MMP-9 in the process of plantar incision-induced postoperative pain, the activity of MMP-9 and the mechanical threshold of mice (WT or MMP-9^−/−^) were measured. As shown in Fig. 1A, compared with the sham group, plantar incision surgery significantly decreased the mechanical threshold of the mice for up to 5 days, and caused severe postoperative pain. Plantar incision surgery increased the activity and the expression of MMP-9 in the spinal cord of mice collected 5 days after the operation (Fig. 1B, C). Interestingly, MMP-9 knockout significantly alleviated postoperative pain compare to the WT + PI group (Fig. 1D). These results suggest that plantar incision surgery could be successfully used to establish a postoperative pain model, and that MMP-9 plays a vital role in the development of postoperative pain.

### ASK1 mediates MMP-9 activation and facilitates the development of postoperative pain

**Fig. 2:**
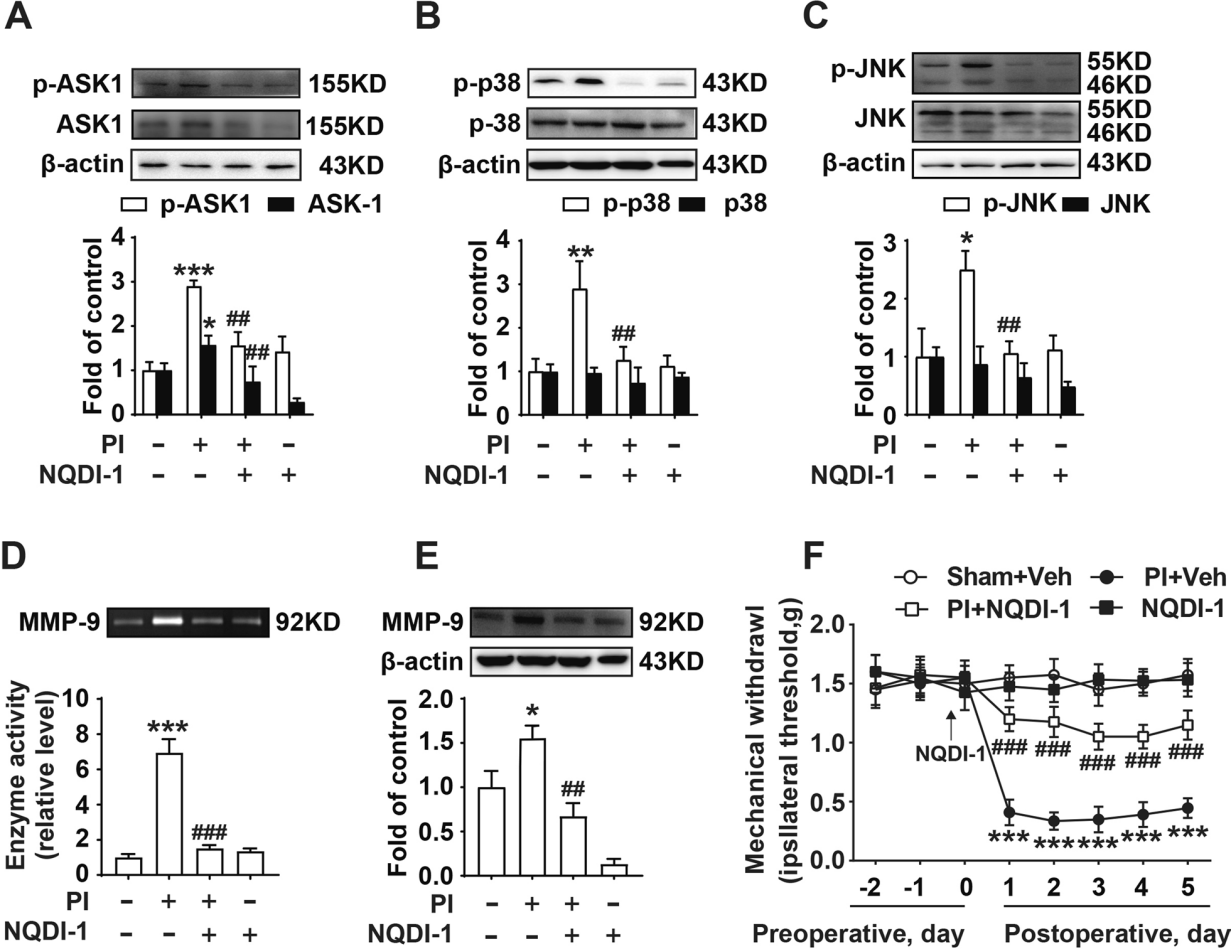
ASK1 inhibition decreased MMP-9 activity and attenuated postoperative pain. The phosphorylation and expression of ASK1 (**A**), p38 (**B**) and JNK (**C**), MMP-9 activity (**D**) and expression (**E**) were measured in the spinal cord of mice treated with the ASK1 inhibitor, NQDI-1 (5 μg/10 μl, i.t./day) (*n* = 6). Lumbar spines (L4–L5) were collected and analyzed 5 days after surgery. **F** Intrathecal injection of the inhibitor, NQDI-1, changed the mechanical threshold of plantar incision surgery (*n* = 12). PI = plantar incision; Veh = vehicle. Significant difference was revealed following one-way ANOVA (A-E), or two-way ANOVA (F) (*P < 0.05, **P < 0.01, ***P < 0.001 vs. sham; ^#^*P* < 0.05, ^##^*P* < 0.01, ^###^*P* < 0.001 vs. PI group; Bonferroni post hoc tests)

Considering the important role of ASK1 in inflammation and apoptosis [12–14], we further explored the mechanism of ASK1 in postoperative pain. As shown in Fig. 2, plantar incision surgery markedly increased the phosphorylation of ASK1, p-38, and JNK, and upregulated the expression of ASK1 but not p-38 and JNK in the spinal cord, while the ASK1 inhibitor, NQDI-1 (5 μg/10 μl, i.t./day, for 5 days), decreased the phosphorylation of ASK1, p-38, and JNK, and reduced the expression of ASK1 (Fig. 2A–C). While considering the influence of ASK1 inhibition on MMP-9, we measured the activity and the expression of MMP-9 in plasma. As shown in Fig. 2D, E, NQDI-1 significantly reduced MMP-9 activity and expression. We also measured the mechanical threshold of mice and found that inhibition of ASK1 increased the mechanical threshold, and thus attenuated postoperative pain (Fig. 2D). These data showed that ASK1 can be used as a target molecule to relieve postoperative pain.

### H_2_ alleviates postoperative pain by decreasing ASK1 phosphorylation and MMP-9 activity in mice

**Fig. 3:**
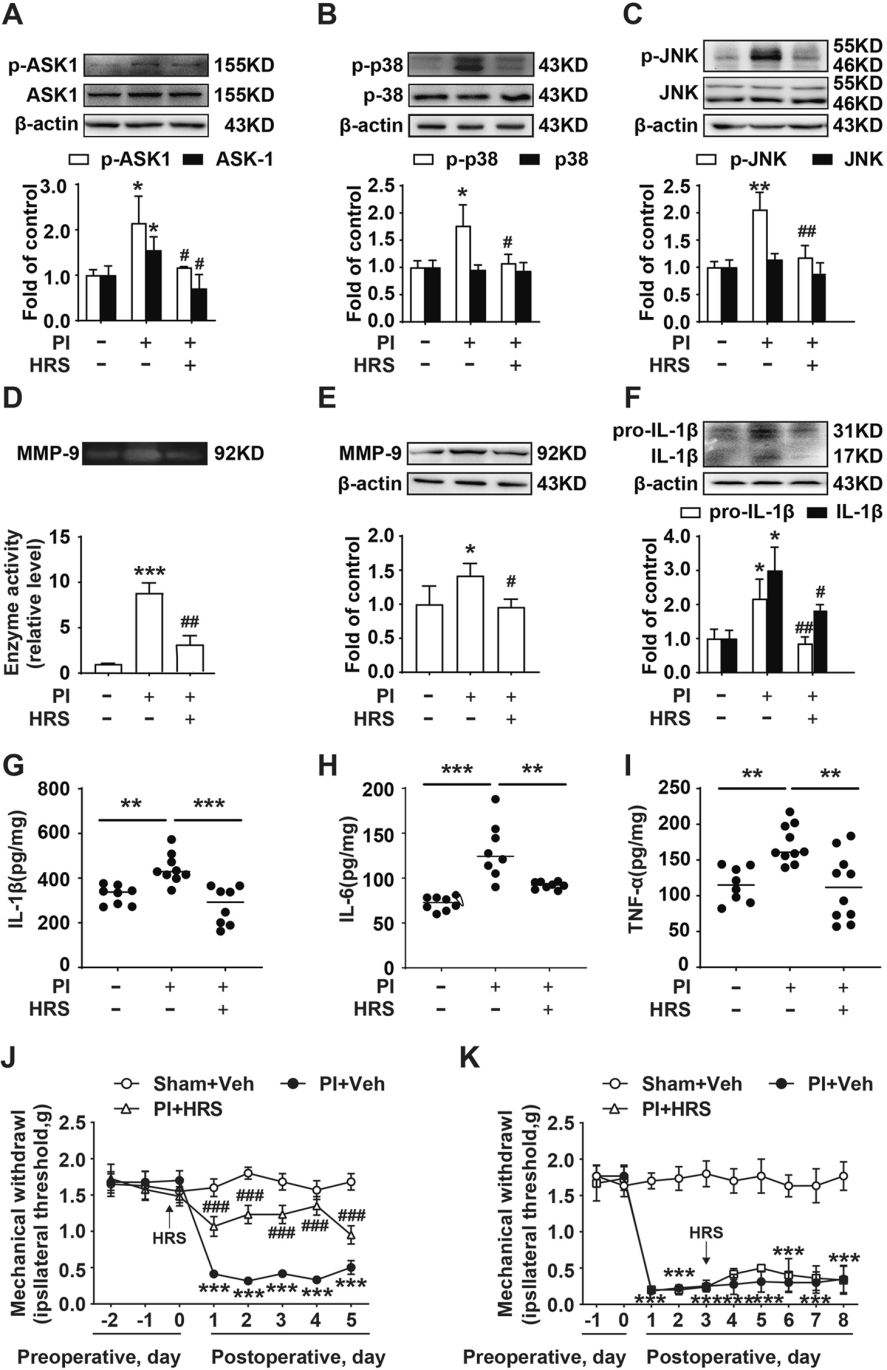
Hydrogen decreased MMP-9 activity and ASK1 phosphorylation to attenuate postoperative pain in mice. Mice were administrated HRS (5 mL/kg, i.p., twice daily), the phosphorylation and expression of ASK1 (**A**), p38 (**B**) and JNK (**C**), the activity and expression of MMP-9 (**D**, **E**), the expression of IL-1β and pro-IL-1β (**F**) were measured using western blot and zymography in the spinal cord (*n* = 6). **G**–**I** The expression of IL-1β, IL-6, and TNF-α were measured using Elisa kit. **J**, **K** Mechanical allodynia after plantar incision surgery was measured using the von Frey test (*n* = 12). Lumbar spines (L4–L5) were collected and analyzed 5 days after surgery. HRS = hydrogen-rich saline; PI = plantar incision; Veh = vehicle. Significant difference is revealed following one-way ANOVA (**A**–**F**) or two-way ANOVA (**G**, **H**) (*P < 0.05, **P < 0.01, ***P < 0.001 vs. sham; ^#^*P* < 0.05, ^##^*P* < 0.01, ^###^*P* < 0.001 vs. PI group; Bonferroni post hoc tests)

Based on the advantages of H_2,_ including rapid membrane translocation to inhibit inflammasome activation, which promotes the maturation of pro-IL-1β and induces inflammation [18, 24], we explored the effects of H_2_ on the ASK1-mediated signaling pathway and IL-1β expression. HRS was injected 6 h before plantar incision surgery. As shown in Fig. 3, HRS mimicked the effects of ASK1 inhibitor, decreasing the phosphorylation of ASK1, p-38, and JNK, and the expression of ASK1 (Fig. 3A-C) compared to the PI group. HRS also decreased the activity and the expression of MMP-9, and inhibited the expression of IL-1β and pro-IL-1β. The Elisa results of IL-1β, IL-6, and TNF-α also verified the role of HRS. To directly investigate the effects of H_2_ on postoperative pain, we measured the mechanical threshold of mice and found that HRS could significantly attenuate mechanical allodynia (Fig. 3J). Additionally, we further examined whether administering HRS after the formation of postoperative pain alleviates postoperative pain. As shown in Fig. 3H, intraperitoneal injection of HRS 3 days after surgery could not significantly attenuate postoperative pain. These data suggested that H_2_ may work as a potential therapeutic agent that could ameliorate postoperative pain.

### H_2_ suppresses the activation of microglia induced by plantar incision surgery in the spinal cord of mice

**Fig. 4:**
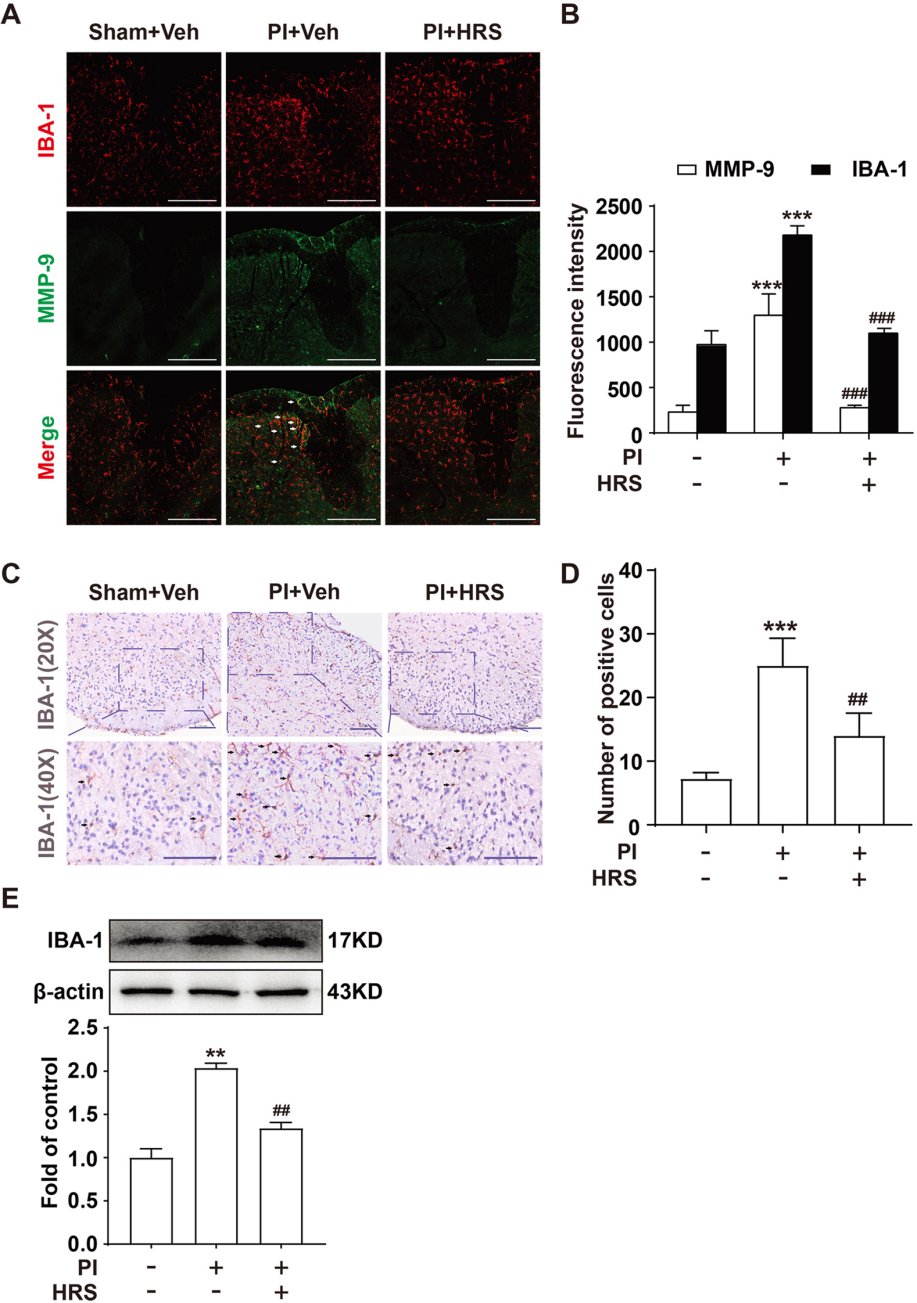
Hydrogen decreased microglia activation that was initially induced by plantar incision surgery in the spinal cord of mice. **A** Confocal images of MMP-9 (green) and its colocalization with microglia (IBA-1, red) in the spinal cord after administering HRS induced by planter incision surgery; *n* = 6. The white arrows indicate IBA-1 colocalization with MMP-9. **B** Immunofluorescence staining analysis after HRS treatment. **C** Immunohistochemistry images of IBA-1 in the spinal cord of the mice and IHC score values of the mice at 5 days; *n* = 4. The black arrows indicate IBA-1. **D** Immunohistochemistry staining analysis after HRS treatment. **E** IBA-1 expression was measured in the spinal cord of mice (*n* = 6). Lumbar spines (L4–L5) were collected and analyzed 5 days after surgery. Scale bar: 200 µm. HRS = hydrogen-rich saline; PI = plantar incision; Veh = vehicle. A significant difference is revealed following one-way ANOVA (*P < 0.05, **P < 0.01, ***P < 0.001 vs. sham; ^#^*P* < 0.05, ^##^*P* < 0.01, ^###^*P* < 0.001 vs. PI group; Bonferroni post hoc tests)

Activation of microglia in the spinal cord could release a large number of proinflammatory factors, such as MMP-9 and IL-1β, resulting in central sensitization and neuropathic pain [8, 25]. Therefore, we measured the expression of IBA-1 (a microglia marker) in the spinal cord using immunofluorescence staining analysis and western blot to investigate the effects of H_2_ on microglia activation. As shown in Fig. 4 A-D, compared with the sham group, plantar incision surgery significantly increased microglial activation in the spinal cord of mice, which was reduced by HRS. The results of western blot also showed that plantar incision surgery increased microglial activation in the spinal cord of mice, which was also decreased by HRS (Fig. 4E). Moreover, further studies found that plantar incision-induced MMP-9 activation was primarily co-labeled with microglia, and HRS could remarkably decrease MMP-9 activity (Fig. 4A, B). These data suggested that H_2_ could reduce plantar incision-induced activation of microglia and MMP-9 in the spinal cord.

### H_2_ increases the expression of thioredoxin in the spinal cord of mice

**Fig. 5:**
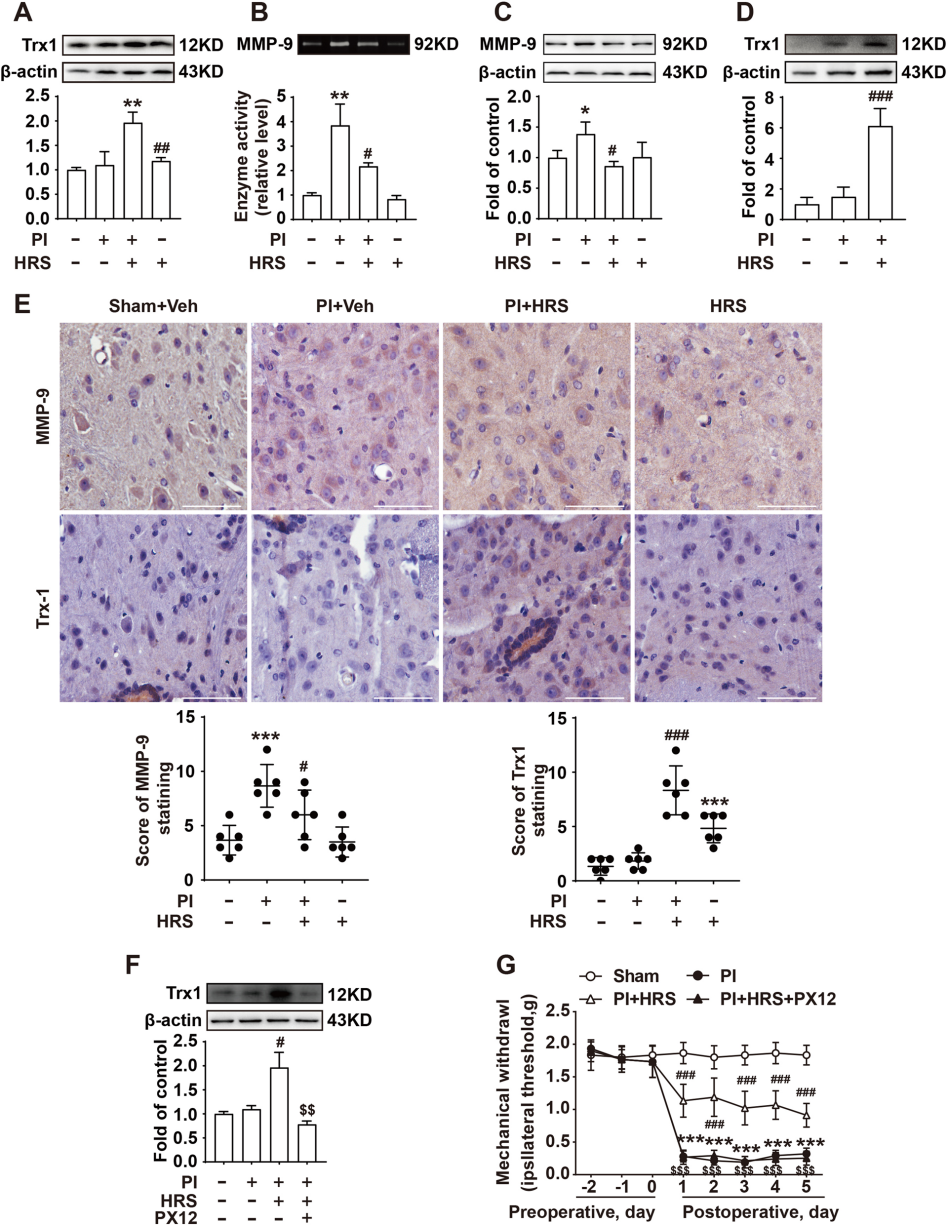
Hydrogen increased Trx1 expression in the spinal cord of mice. The expression of Trx1 in the spinal cord of mice was measured using western blotting at 24 h (**A**) and at 5 days (**D**) after plantar incision surgery (*n* = 6). MMP-9 activity (**B**) and expression (**C**) in the spinal cord of mice after the operation (24 h) were measured (*n* = 6). **D** Immunohistochemistry images of MMP-9 and Trx1 in the spinal cord of mice, and IHC score values of mice at 24 h. Magnification: 400×; *n* = 6. The lumbar spines (L4–L5) are collected and analyzed 24 h after plantar incision surgery. F. Trx1 expression in the spinal cord of mice was measured (*n* = 6). **G** Mechanical allodynia after plantar incision surgery was measured using the von Frey test (*n* = 12). Scale bar: 100um. HRS = hydrogen-rich saline; PI = plantar incision; Veh = vehicle. Significant difference is revealed following one-way ANOVA (**A**–**F**) or two-way ANOVA (**G**) (*P < 0.05, **P < 0.01, ***P < 0.001 vs. sham; ^#^*P* < 0.05, ^##^*P* < 0.01, ^###^*P* < 0.001 vs. PI group; ^$^*P* < 0.05, ^$$^*P* < 0.01, ^$$$^*P* < 0.001 vs. PI + HRS group; Bonferroni post hoc tests)

We measured the related protein level 24 h after plantar incision surgery to further elucidate the early molecular mechanisms of postoperative pain. Since thioredoxin (Trx) promotes ubiquitination and degradation of ASK1 and inhibits ASK1-mediated apoptosis in a redox activity-independent manner [14], the expression of Trx1 was measured, and we found that HRS could significantly increase the expression of Trx1 both at 24 h (Fig. 5A) and 5 days after surgery (Fig. 5D), and could decrease the activity and expression of MMP-9 (Fig. 5B and C) at 24 h in the spinal cord. The results of immunohistochemistry further showed that HRS could increase the Trx1 expression induced by plantar incision surgery in the spinal cord (Fig. 5E). To explore the effect of Trx1 on postoperative pain, we used the of Trx1 inhibitor PX12 and found that PX12 (5 μg/10 μl, i.t./day, for 5 days) could reduce Trx1 expression (Fig. 5F) and abolish the therapeutic effect of HRS on postoperative pain (Fig. 5G). Additionally, we also measured whether HRS could attenuate plantar incision surgery-induced thermal hyperalgesia. We found that HRS could mimick NQDI1 to alleviate thermal hyperalgesia, and Trx1 inhibitor PX12 could abolish the protective effect of HRS (Additional file 1: Fig. S1A and B). These data suggest that Trx1 may be involved in the protection of H_2_ against postoperative pain.

### H_2_ attenuates microglial activation by regulating the Trx1/ASK1/MMP9 signaling pathway in vitro

**Fig. 6:**
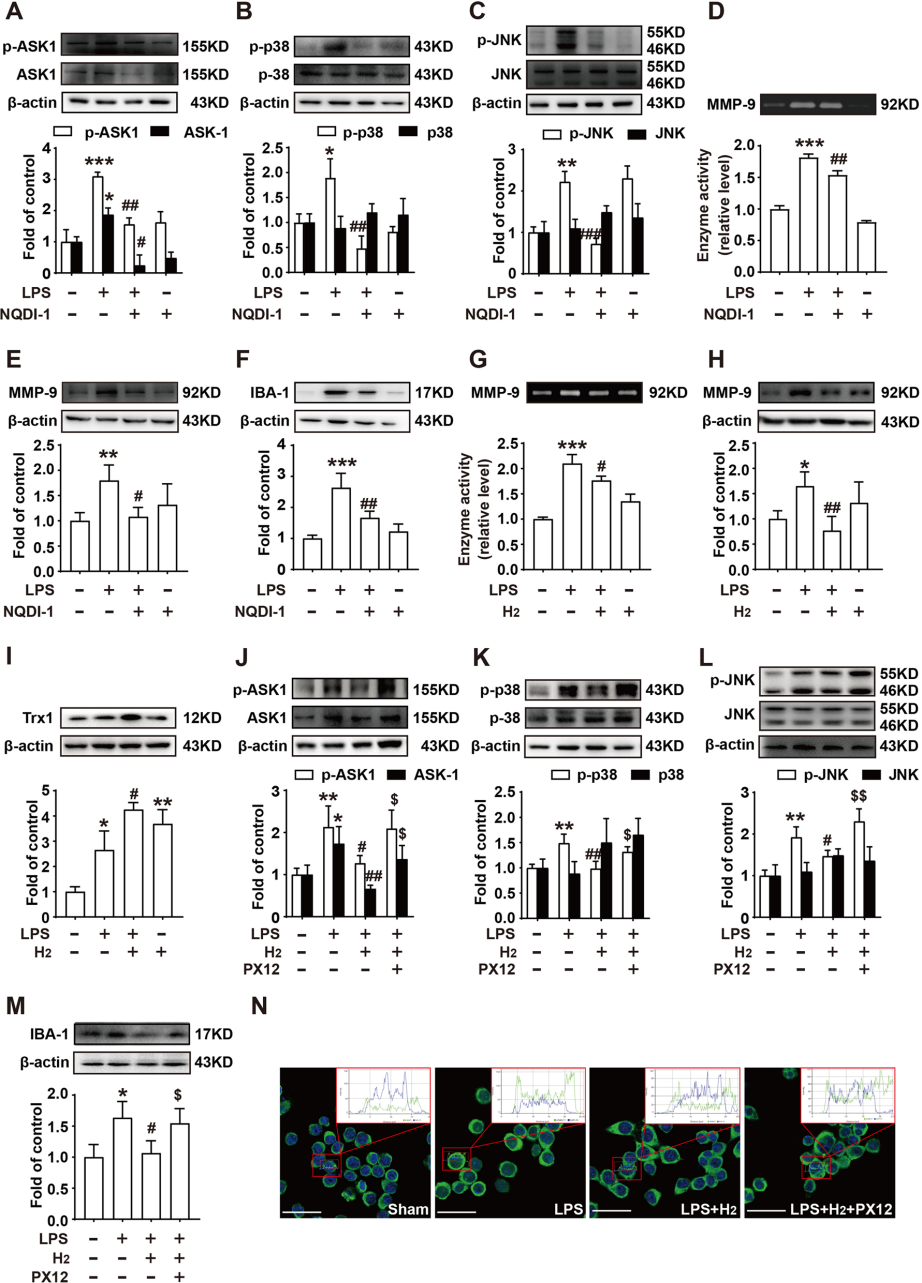
Hydrogen acted on the expression of Trx1/ASK1/MMP9 pathway proteins in vitro. The phosphorylation and expression of ASK1 (**A**), p38 (**B**), and JNK (**C**) were measured using western blot in BV-2 cells after NQDI1 treatment. MMP-9 activity and expression (**D**, **E**), and IBA-1 expression (**F**) were measured using western blot and zymography in BV-2 cells after NQDI1 treatment. The activity of MMP-9 (**G**) and the expression of MMP-9 (**H**) and Trx1 (**I**) were measured after H_2_ treatment. Phosphorylation and expression of ASK1 (**J**), p38 (**K**), and JNK (**L**), and the expression of IBA-1 (**M**) were measured using western blot after H_2_ treatment. **N** Representative images showing NF-kB translocation from the cytosol to the nucleus after different treatments in BV-2 cells. Scale bar: 40 µm; *n* = 4. Cells are collected and analyzed 24 h after H_2_ treatment. Significant differences are revealed following one-way ANOVA (*P < 0.05, **P < 0.01, ***P < 0.001 vs. sham; ^#^*P* < 0.05, ^##^*P* < 0.01, ^###^*P* < 0.001 vs. LPS group; ^$^*P* < 0.05, ^$$^*P* < 0.01, ^$$$^*P* < 0.001 vs. LPS + H_2_ group; Bonferroni post hoc tests)

We further verified the protection mechanism of H_2_ in BV-2 cells in vitro. LPS was used to establish a cellular inflammation model. First, the ASK1 inhibitor, NQDI1, was used to explore the importance of ASK1 inhibition in reducing MMP9 activity. As shown in Fig. 6A-F, compared with the LPS group, NQDI1 decreased the phosphorylation and expression of ASK1, reduced the phosphorylation of p-38 and JNK, decreased the activity and the expression of MMP-9, and inhibited IBA-1 expression in BV-2 cells.

Secondly, we measured the effect of H_2_ and found that H_2_ could mimicked the protective effect of NQDI1. As shown in Fig. 6G, H, and J, compared with the LPS group, H_2_ decreased the phosphorylation and expression of ASK1, and reduced the activity and expression of MMP-9.

Additionally, we investigated the effects of H_2_ on Trx1 upstream of ASK1. As shown in Fig. 6I, H_2_ increased the expression of Trx1 compared to its expression in the LPS group. The Trx1 inhibitor, PX12, was used to assess the importance of TRX1 in vitro. We found that H_2_ could decrease the phosphorylation of ASK1, p-38, and JNK, and reduce the expression of IBA-1 compare to its expression in the LPS group, which were abolished by PX12 (Fig. 6J-M). Furthermore, we measured the NF-κB translocation from the cytosol to the nucleus, as shown in Fig. 6N, and observed that H_2_ significantly inhibited the translocation of p65 induced by LPS, which were abolished by PX12. These data suggested that H_2_ could reduce microglial activation by regulating the Trx1/ASK1/MMP9 signaling pathway in vitro.

## Discussion

In this study, the main findings were as follows: (1) MMP-9 knockout delayed and relieved the development of postoperative pain induced by plantar incision surgery; (2) ASK1 inhibition decreased MMP-9 activity and expression, and attenuated postoperative pain; (3) H_2_ upregulated Trx1 expression and inhibited ASK1/MMP-9 to ameliorate postoperative pain; (4) H_2_ decreased MMP-9 activity via the Trx1/ASK1 signaling pathway and reduced microglial activation in BV-2 cells.

MMPs are involved in the pathogenesis of neuropathic pain. The mechanism of MMPs activation involves modifying cysteine residues (e.g., S-nitrosylation, alkylation, and oxidation) and dissociating the cysteine residue from the zinc-binding site of MMPs [26, 27]. Since the production of proinflammatory factors and cytokines, such as TNF-α, IL-1β, ROS, etc., microglial activation is increasingly implicated. Further oxidation resulted in a stable posttranslational modification with pathological activity, activated MMP-9, and induced apoptosis [28, 29]. Our previous study showed that plantar incisions could increase MMP-9 activity, activate microglia, and promote the development of postoperative pain [11]. In this study, we further showed the importance of MMP-9 in the development of postoperative pain using MMP-9 knockout mice (Fig. 1D). Furthermore, MMP-9 co-localized with microglia IBA-1 and aggravated the cleavage of IL-1β (Fig. 4A). We also found that inhibition of ASK1 could ameliorate pain and reduce MMP-9 activity (Fig. 2), suggesting that MMP-9 and its upstream ASK1 are associated with the facilitation of postoperative pain. Regulation of ASK1/MMP9 in microglia may receive considerable attention as a potential therapeutic target for ameliorating postoperative pain.

HRS and H_2_ have been confirmed to alleviate hyperpathia and activate autophagy in neuropathic pain models [24, 30]. In this study, HRS was used for the first time to treat postoperative pain. In accordance with the effects of H_2_ on p-ASK1, p-p38, and p-JNK (Fig. 3A-C), H_2_ decreased MMP-9 activity and expression, inhibited the expression of IL-1β, IL-6, and TNF-α (Fig. 3D-I), and effectively attenuated incision-induced postoperative pain (Fig. 3J).

Furthermore, we explore how H_2_ regulates the phosphorylation of ASK1, p38, and JNK. Trx1 is an endogenous 12 kDa multifunctional protein with two redox-active half-cysteine residues -Cys-Gly-Pro-Cys- [31]. It has been identified in all living cells and is related to cell proliferation and apoptosis, where it is responsible for protecting cells from oxidative stress by scavenging ROS [31, 32]. Experimental results show that intravenous administration of recombinant human thioredoxin and overexpression of Trx1 in transgenic mice confer resistance to ROS-induced cell death, ultimately decreasing brain damage in cerebral ischemia models [33, 34]. Trx1 overexpression extends antioxidant protection, attenuates mitochondrial damage, and prolongs survival during sepsis [35]. Furthermore, along with the decrease in Trx1 level, NLRP3 expression increases inflammation in injured tissue [36]. Since Trx1 exerts its role by interacting with its binding protein, ASK1, it inhibits the activation of ASK1 [14]; moreover, our data showed that H_2_ could increase the expression of Trx1 (Fig. 5A, D, E) and attenuate postoperative pain, which was abolished by the Trx1 inhibitor PX12 (Fig. 5G), these data indicate that Trx1 is an endogenous neuroprotective protein that is involved in proves through which H_2_ reduces postoperative pain. To further investigate the protection effects of H_2_, we collected and analyzed BV-2 cells after H_2_ treatment. We found that H_2_ could mimic the ASK1 inhibitor NQDI1 as it decreases the phosphorylation of ASK1, p38, and JNK, and reduces MMP-9 activity and expression (Fig. 6A–F). We also found that the protective effects of H_2_ were abolished by the Trx1 inhibitor, PX12, in BV-2 cells (Fig. 6G–N). These data demonstrate that H_2_ could attenuate postoperative pain by regulating the Trx1/ASK1/MMP-9 signaling pathway, and provides further details regarding the mechanism of H_2_ therapy.

H_2_ has been shown to be safe with few adverse effects. Compared to vitamin E and superoxide dismutase, H_2_ is a selective antioxidant that reduces cytotoxic oxygen radicals [18]. Inhalation of H_2_, drinking hydrogen water, injection of hydrogen saline, and direct incorporation of molecular hydrogen by diffusion, including eye drops, baths, and cosmetics, are the main methods of ingesting or consuming H_2_ [19]. Inhalation of H_2_ suppresses not only the initial brain injury, but also its progressive damage [18]. H_2_ has beneficial effects including the promotion of microglia M2 polarization and the reduction of inflammation [20, 23]. Interestingly, oral administration of hydrogen water was reported to alleviate neuropathic pain in mice by reducing oxidative stress. Since oxidative stress injury is an important pathological mechanism of postoperative pain [37], oral administration of hydrogen water may also be useful in attenuating postoperative pain. The oral pathway is more conducive to the promotion of the clinical application of H_2_.

One of the limitations of the research is that we did not consider gender differences in H_2_ therapy. We only considered the response of male mice to postoperative pain, while ignoring the difference in magnetic responses to pain. We will explore this in future research.

## Conclusions

**Fig. 7:**
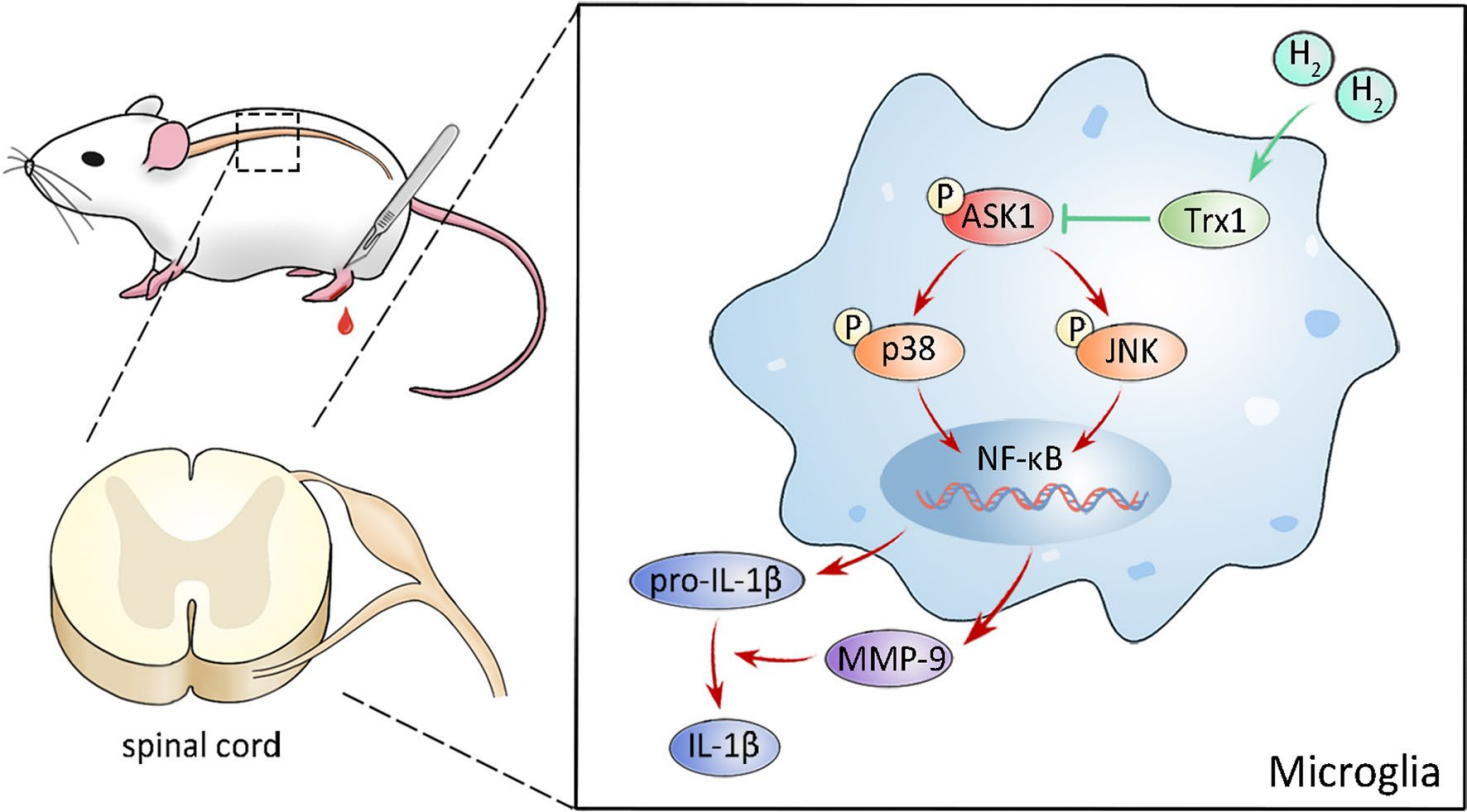
H_2_ protects against plantar incision-induced postoperative pain by upregulating the Trx1/ASK1/MMP-9 signaling pathway. Plantar incision surgery increases ASK1/JNK/p38 phosphorylation mediated microglia activation, enhances MMP-9 activity and IL-1β cleavage, and subsequently induces neuroinflammation, which contributes to the progression of postoperative pain in mice. Inhalation of H_2_ and administration of HRS, which increases Trx1 expression, could ameliorate postoperative pain

In summary, we demonstrated that H_2_ attenuates postoperative pain induced by plantar incisions by inhibiting the ASK1/JNK/p38/MMP9 signaling pathway and microglial activation, and this may be related to Trx1 (Fig. 7). Our findings suggest that H_2_ may be a potential drug for the treatment of postoperative pain.

## Data Availability

The datasets during and/or analyzed during the current study are available from the corresponding author on reasonable request.

## Abbreviations

MMPs: Matrix metalloproteases
ASK1: Apoptosis signal-regulated kinase 1
IL-1β: Interleukin-1β
TNF-α: Tumor necrosis factor alpha
ERK: Extracellular signal-regulated kinase
JNK: C-Jun N-terminal kinase
Trx1: Thioredoxin
•OH: Hydroxyl radical
ONOO-: Peroxynitrite
HRS: Hydrogen-rich saline
H2: Hydrogen gas
LPS: Lipopolysaccharide
DMSO: Dimethyl sulfoxide
IBA-1: Anti-ionized calcium-binding adaptor protein 1
NLRP3: Nod-like receptor family pyrin domain containing protein 3
